# Impact of repeated exposure to CPM on CPM efficiency and pain sensitivity in healthy adults: a randomized controlled trial

**DOI:** 10.3389/fpain.2025.1677563

**Published:** 2025-11-13

**Authors:** Priyanka Rana, Michael E. Robinson, Meryl J. Alappattu, Joseph Riley III, Donovan Lott, Mark D. Bishop

**Affiliations:** 1 Department of Physical Therapy and Movement Science, University of Texas at El Paso, El Paso, TX, United States; 2 Department of Clinical and Health Psychology, University of Florida, Gainesville, FL, United States; 3 Center for Pain Research and Behavioral Health, University of Florida, Gainesville, FL, United States; 4 Department of Physical Therapy, University of Florida, Gainesville, FL, United States; 5 Pain Research and Intervention Center of Excellence, University of Florida, Gainesville, FL, United States; 6 Department of Community Dentistry and Behavioral Science, University of Florida, Gainesville, FL, United States

**Keywords:** pain, physical therapy, rehabilitation, musculoskeletal disorder, exercise

## Abstract

Conditioned pain modulation (CPM) is a behavioral measure of diffuse noxious inhibitory control (DNIC), an endogenous central pain modulatory mechanism in which one pain stimulus suppresses the perception of another. CPM efficiency is reduced in individuals with chronic pain and serves as a potential predictor for the development of chronic pain conditions. Current research indicates that CPM, traditionally viewed as a static metric, may exhibit protocol-dependent variability in its effects on pain sensitivity, potentially through neuroplastic mechanisms and central pain processing pathways. This randomized controlled trial (NCT05783362) investigated whether repeated activation of central pain modulatory systems enhances CPM efficiency. The secondary aim examined associations between repeated CPM exposure and pain-related psychological factors. Sixty healthy participants (52% female; ages 18–75) were randomly allocated to High Exposure (HE), Low Exposure (LE), or No Exposure (NE) CPM intervention groups. Pre- and post-intervention measures included CPM efficiency and pain sensitivity across thermal and pressure pain tests. Two-way ANOVA analyses revealed significant main effects for both time (*p* < 0.001, *η*^2^ = 0.23) and intervention (*p* = 0.030, *η*^2^ = 0.107) on CPM efficiency when comparing HE and LE groups from pre- to post-intervention. One-way ANOVA analysis at the final visit showed that HE demonstrated significantly higher CPM efficiency compared to LE (*p* = 0.02, Cohen's *d* = 0.73), while comparisons between HE and NE approached but did not reach statistical significance (*p* = 0.053–0.060; medium-to-large effect sizes, Cohen's *d* > 0.70). This was supported by increased heat threshold pain intensity ratings (*p* < 0.001, *η*^2^ = 0.13), suggesting broader adaptations in pain processing that strengthen descending pain control mechanisms. Other QST measures and psychological variables remained unchanged, suggesting the specificity of the modulatory enhancement. Results support the plasticity of endogenous pain modulation and suggest potential therapeutic applications for pain management interventions.

**Clinical Trial Registration:**

https://clinicaltrials.gov/study/NCT05783362, identifier NCT05783362.

## 1 Introduction

The complex integration of pronociceptive and antinociceptive neural systems fundamentally underlies pain perception (1), with quantitative sensory testing (QST) providing mechanistic insights into these processes (2–4). Impaired endogenous antinociceptive capacity, determined using QST, is linked to chronic pain conditions (5–7). Conditioned pain modulation (CPM) represents a behavioral measure of dynamic QST that reflects aspects of diffuse noxious inhibitory control (DNIC), wherein one noxious stimulus suppresses perception of another, quantifying individual endogenous pain regulatory capacity (8–10). CPM operates through conditioning stimulus-induced inhibition of a secondary test stimulus (9, 11), with the magnitude of test stimulus response change defining CPM “efficiency”.

Clinical investigations have demonstrated that a subset of individuals with chronic pain exhibit significantly attenuated CPM efficiency compared to healthy controls (5, 12–14). Notably, therapeutic interventions demonstrate concurrent improvements in CPM efficiency and clinical outcomes in patients with osteoarthritis and diabetic neuropathy (15, 16). Clinical evidence demonstrates enhanced analgesic efficacy of duloxetine in patients with diabetic neuropathy and impaired CPM (17), while surgical interventions for osteoarthritis via total hip arthroplasty demonstrate restoration of CPM efficiency (16). Comparative analyses consistently reveal greater CPM efficiency in healthy populations vs. patients with chronic pain, with CPM enhancement correlating with clinical pain reduction (9, 18). These findings suggest interventions that improve endogenous inhibitory modulation of pain, as measured using CPM, should be considered in managing chronic pain.

Exercise represents a key intervention modulating endogenous pain control through exercise-induced hypoalgesia (EIH), wherein painful exercise activates descending inhibitory pathways producing subsequent analgesia (19, 20). EIH demonstrates neuroplastic capacity, with systematic training producing progressive adaptations in pain sensitivity (21–23). For example, pressure pain ratings decreased after exercise that was perceived as painful for both young and older adults (24). Athletic populations exhibit elevated pain thresholds and enhanced pain tolerance compared to sedentary controls, attributed to repetitive nociceptive exposure during high-intensity training (25). These adaptations suggest structural and functional modifications within central pain processing circuits through repeated activation (22, 26, 27). Conditioned pain modulation (CPM) and EIH share common neurophysiological mechanisms involving endocannabinoid, opioid, and serotonergic systems (28, 29). Given EIH's demonstrated plasticity, we hypothesized that repeated activation of central mechanisms through CPM may similarly induce neuroplastic enhancements in inhibitory function, which might in turn alter pain sensitivity.

Pain perception is modulated by emotions through shared descending pathways originating from cerebral structures involved in affective and sensorimotor processing (30, 31). Since conditioned pain modulation (CPM) utilizes these same descending inhibitory pathways, psychological factors may influence CPM efficiency through reciprocal interactions. Supporting this hypothesis, Lazaridou et al. (32) demonstrated elevated back pain intensity in participants with concurrent high depression and low CPM efficiency, potentially mediated by depression-induced alterations in descending modulatory brain regions (32). However, other studies have found no significant association between anxiety and CPM efficiency (33, 34). Additionally, Ibancos-Losada et al. (33) reported no associations between CPM and depression, catastrophizing, pain history, or pain tolerance in healthy individuals (33). The predominantly cross-sectional nature of these studies, with single-timepoint CPM assessments, contributes to inconsistent findings regarding psychological factor-CPM relationships.

Therefore, the primary goals of this study to determine the extent to which repeated exposure to CPM protocols, i.e., “training” CPM, modifies: (1) static QST measures of pain sensitivity (thermal and pressure pain thresholds and tolerance), with an emphasis on (2) dynamic QST measures of endogenous pain modulation (CPM efficiency). A secondary aim examined whether repeated CPM exposure affects pain-related psychological factors. We hypothesized that endogenous pain modulatory mechanisms would demonstrate differential changes in efficiency based on exposure intensity, with participants receiving high-frequency CPM exposure demonstrating reduced pain sensitivity (measured by thermal and pressure pain thresholds) and enhanced endogenous pain inhibition (measured by CPM efficiency) compared to those receiving low or no exposure. In addition, we hypothesized that repeated CPM protocol exposure would alter pain-related psychological factors.

## 2 Methods

### 2.1 Study design

The present study was a randomized controlled trial. The study was approved by the University of Florida's Institutional Review Board (IRB 202300187) and registered with the ClinicalTrials.gov (NCT05783362).

### 2.2 Participants

Sixty pain-free male (48.33%) and female (51.67%) participants between 18 and 75 years of age were recruited via social media and posted advertisements at the University of Florida and in the surrounding Gainesville, Florida community. A study staff member contacted individuals who expressed interest via an IRB-approved phone script or an IRB-approved e-mail script to provide an overview of basic information about the study and answer screening questions. Eligible participants were invited to schedule an initial session. All the subjects received oral and written information about the experiment and gave written consent before participating in the study.

Participants were excluded if they met any of the following criteria: (a) non-English speaking; (b) systemic medical condition is known to affect sensation (i.e., diabetes)/ conditions that require prescribed medical treatment; (c) regular use of prescription pain medication to manage pain; (d) current or history of chronic pain condition; (e) currently using blood thinning medication; (f) any blood clotting disorder such as hemophilia; (g) any contraindication to the application of ice or cold packs, such as uncontrolled hypertension, cold urticaria, cryoglobulinemia, paroxysmal cold hemoglobinuria, and circulatory compromise (h) engagement in vigorous physical activities like heavy lifting, digging, aerobics or fast bicycling for two or more days a week; (i) have pain every day or most of days; (j) getting any pain treatment.

All participants were instructed to refrain from any exercise throughout the 2-week study period.

Handedness was assessed for all participants. Fifty-nine participants (98.3%) were right-handed, and one participant (1.7%) was left-handed. This distribution minimized potential confounding from overlapping stimulus application sites, as the CPM conditioning stimulus was consistently applied to the left hand while other quantitative sensory testing was performed on the dominant side.

### 2.3 Measures

#### 2.3.1 Quantitative sensory testing

Pain responses to all QST protocols were recorded using the numeric pain rating scale (NRS). The numeric pain rating scale is a 101-point scale (0–100) in which patients verbally rate their pain from 0, indicating “no pain”, to 100, indicating “the most intense pain sensation imaginable”. The NRS demonstrates excellent psychometric properties with high test-retest reliability (ICC = 0.95) and strong correlation with visual analog scales (r = 0.94), and has been validated for acute and chronic pain assessment (35–37).

##### 2.3.1.1 CPM-outcome (assessment protocol)

**Figure 1:**
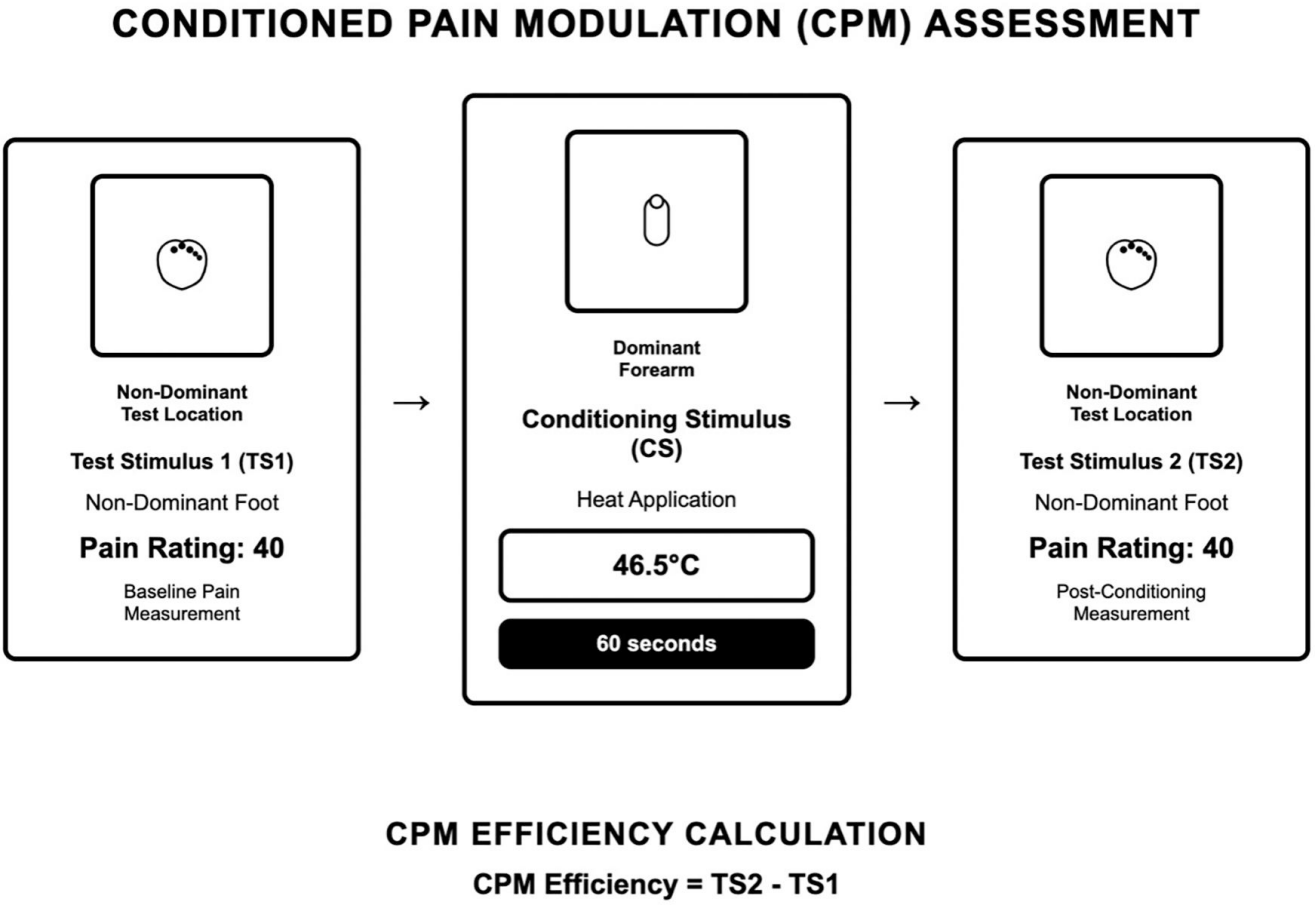
CPM-outcome protocol (assessment).

As demonstrated in Figure 1, participants received a **suprathreshold pressure pain stimulus (Pain-40)** applied to the web space of the dominant foot. Participants were instructed to say “stop” or “pain” so the stimulus could be terminated “when you feel pain equal to 40 out of 100”. Pressure was applied per ascending intensity at 1 kg.s^−1^ until the pain reached 40 out of 100, then discontinued. This was repeated two times, and the average was analyzed (1). Participants then received a conditioning stimulus contact heat stimulus applied to the thenar of the left hand for 60 s at an intensity of 46.5°C (38). Participants were asked to rate the heat pain during a 60 s trial. Participants were instructed that they could remove their hands at any time if the heat was intolerable. After 60-seconds, the contact heat was completely removed, and the **suprathreshold pressure pain stimulus (Pain-40)** was re-applied to the web space of the dominant foot. Conditioned pain modulation was calculated as the average **pressure pain-40 threshold (kg)** of the first testing stimulus series minus the average pressure pain-40 threshold (kg) of the second testing stimulus series. Negative numbers indicate efficient pain modulation (17). CPM served as our primary measure of endogenous inhibitory capacity.

##### 2.3.1.2 Pressure pain threshold

An algometer (AlgoMed, Ramat Yishai, Israel) with a 1 cm diameter rubber tip was applied at 1 kg.s^−1^ to the dominant hand at the first dorsal interosseous muscle. Participants were instructed to indicate when the sensation first changes from pressure to pain (pain threshold). This procedure was repeated two times, and the average pressure to threshold was analyzed.

##### 2.3.1.3 Thermal threshold and tolerance

A slowly ramping thermal stimulus was delivered to the skin of the participant's dominant forearm using a computer-controlled TSAII NeuroSensory Analyzer from Medoc, Inc. Thermal stimuli increased from a baseline of 35°C to a maximum of 51°C in ascending one-degree intervals. Participants were instructed to indicate when the sensation first changes from warmth to pain **(pain threshold)** and when the sensation becomes “so painful you can no longer tolerate it” **(pain tolerance)**. This procedure was repeated two times and the average temperature to achieve the threshold and to achieve tolerance was calculated.

##### 2.3.1.4 After sensations

The participants were asked to rate the magnitude of their pain sensation following the removal of the thermode. The investigator cued the participants to rate their pain every 15 s. These ratings were obtained for 60 s. Aftersensations are thought to reflect endogenous inhibitory capacity (39) and served as our secondary measure of endogenous inhibitory capacity.

#### 2.3.2 Psychological factors

##### 2.3.2.1 The brief pain inventory- short form (BPI-sf)

The Brief Pain Inventory—Short Form (BPI-sf) is a 9 item self-administered questionnaire used to evaluate the severity of a pain and the impact of this pain on daily functioning. The pain severity score is calculated from the four items about pain intensity. Each item is rated from 0, no pain, to 10, pain as bad as you can imagine, and contributes with the same weight to the final score, ranging from 0 to 40 (40). This instrument demonstrates established psychometric properties of reliability and validity for the assessment of pain intensity and functional interference across diverse populations, including both community-dwelling individuals and clinical cohorts presenting with chronic pain conditions (Cronbach α: 0.87) (41).

##### 2.3.2.2 Center for epidemiological studies: depression scale (CES-D)

The CES-D is a 20-item measure of symptoms of depression. It scored from 0 to 60 and higher scores indicate the presence of more symptomatology. This instrument demonstrates established psychometric properties of reliability and validity for the comprehensive assessment of depressive symptomatology across multiple domains, including affective, cognitive, behavioral, and somatic manifestations, in both community-based and clinical populations (Cronbach α = 0.88) (42, 43).

##### 2.3.2.3 Positive and negative affect schedule (PANAS)

The PANAS is a 20-item scale measuring both positive and negative affect in which items scored from 10 to 50. Each item indicates the extent to which the respondent has felt this way in the indicated time frame. The instrument has psychometric indicators of internal consistency and validity for the assessment of affective valence, specifically measuring both positive affect and negative affect constructs in diverse populations including normative samples and individuals presenting with clinical conditions (Cronbach α = .91 for the PANAS-P and.87 for the PANAS-N) (42, 44, 45).

##### 2.3.2.4 Generalized anxiety disorder-7 (GAD-7)

The GAD-7 is a 7-item questionnaire in which scoring is calculated by assigning scores of 0, 1, 2, or 3 to the response categories, respectively, of “not at all”, “several days”, “more than half the days”, and “nearly every day”. GAD-7 total score ranges from 0 to 21. Higher scores indicate more significant anxiety. It is a valid and reliable measure for assessing anxiety symptoms in the general population, including healthy individuals (Cronbach α = 0.895) (46, 47).

##### 2.3.2.5 Fear of pain questionnaire (FPQ-9)

The FPQ-9 is a 9-item questionnaire in which individuals respond to each item on a five-point scale (scored from 1 to 5) from “not at all” to “extreme”. Total scores range from 5 to 45, with higher scores indicating greater fear of pain. The tool demonstrates acceptable psychometric indicators for the reliable measurement of fear and anxiety specifically related to pain experiences in healthy participant populations (Cronbach α = 0.873) (48, 49).

##### 2.3.2.6 Expectation

Individuals were asked if they expected to decrease or increase pressure pain associated with the CPM measurement post-intervention using a NRS scale ranging from 0 to 100 (0, “No pressure pain at all” to 100, “Most intense pressure pain”). This is consistent with the NRS format used for actual pain ratings throughout the study. This approach maintains methodological consistency by using the same scale for both expected and experienced pain intensity.

#### 2.3.3 CPM-intervention (training protocol)

**Figure 2:**
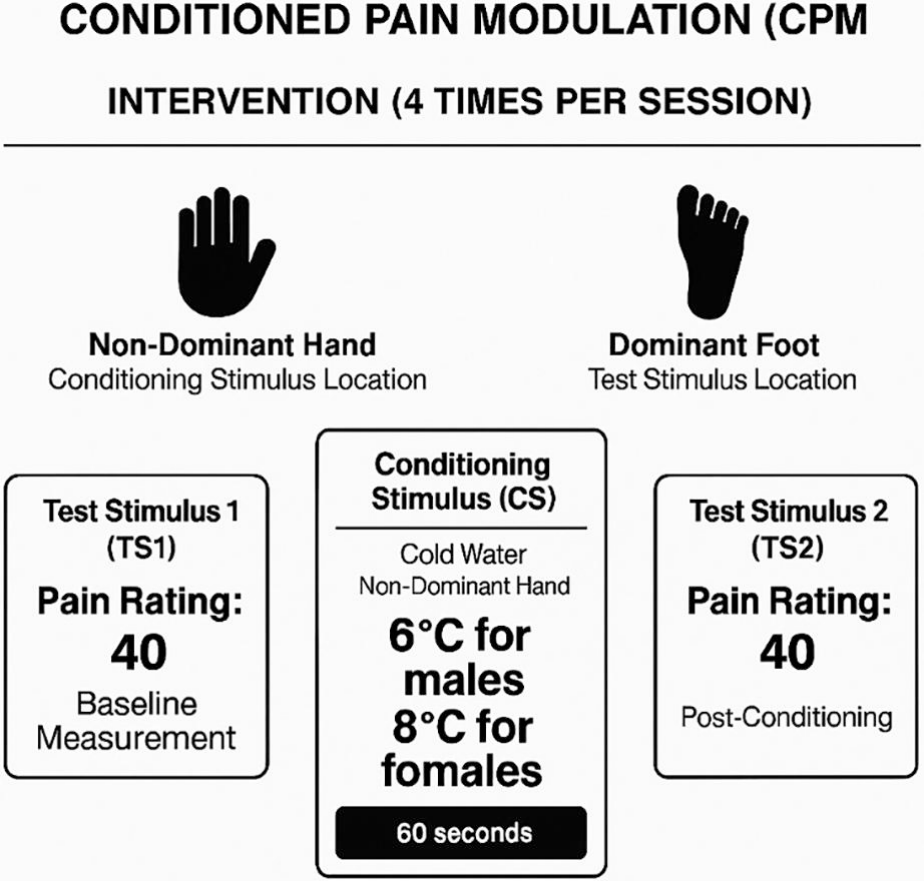
CPM-intervention protocol (training).

To separate potential practice effects, we used different conditioning and test stimuli and body parts for CPM as the intervention, compared to the CPM assessment. As shown in Figure 2 participants received a suprathreshold pressure pain stimulus (Pain-40) applied to the web space of the dominant foot. Pressure was applied per ascending intensity at 1 kg.s^−1^ until the participant's pain intensity reached 40 out of 100, then discontinued. This testing stimulus was repeated twice, and the average was calculated (1). Participants then received a conditioning stimulus by immersing non-dominant hand into the water cooled by a refrigeration unit (NESLAB RTE 7 Digital One, Thermo Scientific Co., Massachusetts, USA) that circulates water continuously to maintain a constant temperature of 6°C (males) or 8°C (females) for 60 s based on prior literature demonstrating sex differences in cold pain sensitivity, with women generally showing lower tolerance to cold pain (50, 51). Subjects were asked to rate the cold pain during the four 60 s trials. Subjects were instructed that they could remove their hand at any time if the water is intolerable. If this occurred, or if subjects rated the pain higher than 50 (0–100 scale), the bath temperature was increased for the subsequent trial. If the ratings were less than 20, a small about of ice was added to lower the temperature by up to 4°C. This individualized adjustment procedure ensured consistent pain intensity across participants regardless of sex. Participants completely removed their hand from the cold pressor for 30 s following each of the four 60 s immersions, during which time the suprathreshold pressure pain stimulus (Pain-40) was re-applied per the sequential paradigm to the web space of the foot. Participants completed four 60 s periods of immersion. Conditioned pain modulation was calculated as the average pressure pain-40 threshold (kg) of the first testing stimulus series minus the average pressure pain-40 threshold (kg) of the second testing stimulus series. Negative numbers indicated efficient pain modulation (17).

#### 2.3.4 Participant allocation and experimental design

**Figure 3:**
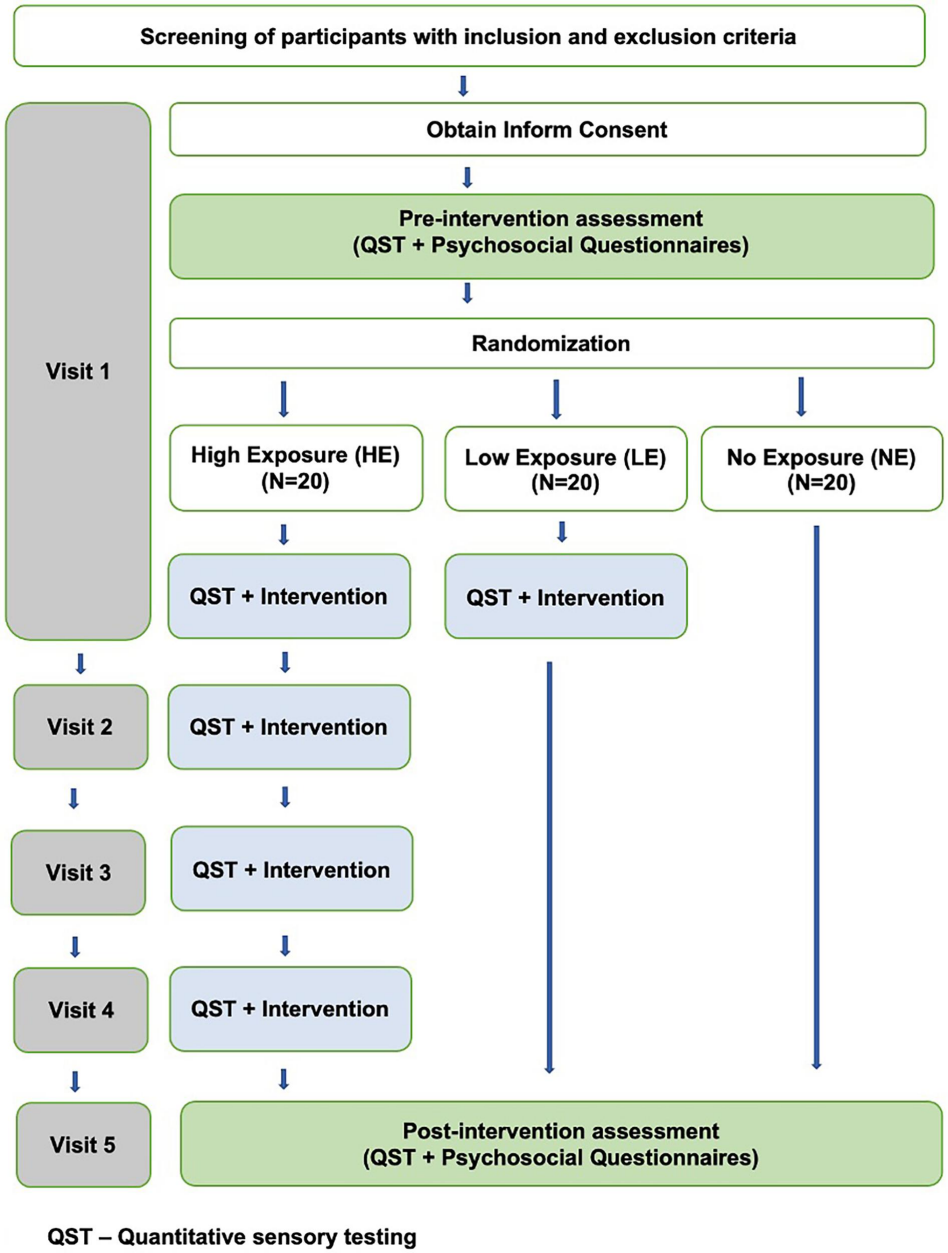
Study procedure.

Participants were randomly assigned intervention groups using a computerized allocation sequence, which was securely stored in a password-protected digital repository to maintain allocation concealment throughout the study protocol. Participants were randomly assigned to three groups using computer-generated randomization: High Exposure (HE), Low Exposure (LE), and No Exposure (NE) as shown in Figure 3.

##### 2.3.4.1 HE

Participants in this group received five sessions in total. Four of these sessions involved the Intervention, while the first and fifth sessions included questionnaires and QST as an outcome measure. Each session was scheduled every 48–72 h over two weeks.

##### 2.3.4.2 LE

Participants in this group received two sessions in total. This included one session of the Intervention, with questionnaires and QST as outcome measures administered in the first and fifth sessions. The final session was after the two weeks of the first session.

##### 2.3.4.3 NE

The NE group served as a true “no exposure” group that did not have any experimental engagement of CPM. Our rationale for this design was that even baseline testing could constitute “exposure” to the “intervention” essentially resulting in two low exposure groups. Participants completed two sessions involving questionnaires and one QST session, with QST conducted exclusively during the final session after two weeks of the first session.

#### 2.3.5 Experimental sessions

Participants who consented were signed up for individual blocks of testing time (90 min) based on group assignment as illustrated in Figure 3. Measures were collected by the primary investigator, co-investigator, and/or research assistant under the direct supervision of the primary or co-investigator, with no blinding among research personnel; however, participants remained blinded to their group allocation.

Psychological measures were administered at both baseline and final visits. At every session, participants completed the expectation and anxiety about testing, and blood pressure was assessed with a digital blood pressure monitor before quantitative sensory testing. Participants with readings exceeding 140/90 mmHg were excluded from the session and informed of their elevated blood pressure. Next, a baseline assessment of pain sensitivity using QST (slow ramp, after-sensations, pressure pain threshold upper extremity, and CPM-Outcome) was completed. After testing, participants sat quietly for 15 min to allow changes in pain sensitivity to normalize after QST (5). Those assigned to the HE or LE groups then completed the CPM-Intervention protocol.

## 3 Analysis

The sample size was determined using G power based on a reduction in psychophysical pain modulation using effect sizes for changes in CPM efficiency from a study comparing massage to conditioned pain modulation (52). We used a large effect size (eta2 = 0.17), a two-tailed null hypothesis, and an alpha of 0.05 to generate a conservative power estimate. Fifteen participants per treatment group would provide greater than 80% power to detect a 2 (Time) × 2 (Intervention: HE vs. LE) mixed ANOVA design. This 2 × 2 design (rather than 2 × 3) reflects our two-step analytical approach: the NE group received no baseline CPM assessment, as we hypothesized that even a single baseline CPM test could constitute meaningful “exposure” to pain modulatory mechanisms. Our sample size (*n* = 60; 20 per group) exceeded the minimum required (*n* = 44 for 2 × 2 design) and provided adequate power for both our primary mixed ANOVA and secondary between-groups comparisons.

The data were analyzed using R and were initially checked for outliers, which were defined as values greater than 3 standard deviations from the mean of the respective variable (e.g., CPM efficiency, QST measures). Parametric assumptions were evaluated using various diagnostic procedures, including visual assessment of the histogram to assess conformity to the normal distribution, examination of z ratio skewness and kurtosis statistics, application of the Shapiro–Wilk test, and utilization of the Kolmogorov–Smirnov test for assessing normality. If, among the four diagnostic tests employed, at least three indicated conformities to the normal distribution, the data were considered to exhibit normality. Descriptive statistics were computed for demographic variables, psychological factors, and medical history. Continuous variables were reported as means ± standard deviations), while categorical variables were expressed as frequencies (percentages). Demographic and psychological variables were assessed for balance across intervention arms using one-way ANOVA for continuous variables and chi-squares for categorical variables. Chi-square analysis confirmed balanced sex distribution across intervention arms [*χ*^2^(2) = 0.87, *p* = 0.65].

For analysis purposes on CPM, we followed recommendations (9) on presenting results and calculation of CPM using the difference between the pressure pain-40 threshold before the application of the conditioning stimulus (pre-CPM) minus the pressure pain-40 threshold after the application of the conditioning stimulus (post-CPM). The CPM-intervention protocol (pressure pain-40 test stimulus with cold water conditioning stimulus; see Figure 2) was used for training purposes only and was not included in outcome analyses.

We examined the impact of the intervention on CPM efficiency using two steps. The NE group served as a true “no exposure” group that did not have any engagement of CPM during the experimental procedures. This necessitated a two-step process to examine changes in CPM efficiency in the intervention groups.

First, two-way Analysis of Variance (ANOVA) was used to assess the relationship between QST variables, Intervention arm (HE and LE) and Time (pre-intervention, post-intervention), under the assumption of a normal distribution in the dependent variables. After the ANOVA, *post-hoc* comparisons were carried out using Tukey's procedure to discern specific group differences. The effect size (ES) was estimated using eta squared (*η*^2^) as a measure of the magnitude of the observed effects. In cases where dependent variables violated normality assumptions, a nonparametric approach utilizing Aligned Rank Transform (ART) ANOVA was implemented to examine relationships between intervention arms (HE and LE) and QST variables. Subsequent to significant ART ANOVA findings, *post-hoc* comparisons were performed using estimated marginal means (emeans) to determine significant differences between specific group pairs.

Next, a one-way analysis of variance (ANOVA) was conducted to examine between-group differences across all three intervention arms (HE, LE, and NE) on QST outcome variables at the post-intervention assessment point. In instances where the dependent variables deviated from a normal distribution, a nonparametric test, specifically the Kruskal–Wallis *H*-test, was employed to assess the relationship between the Arms (HE, LE, and NE) and pain sensitivity. After the Kruskal–Wallis test, *post-hoc* comparisons were conducted utilizing the Mann–Whitney *U*-test to pinpoint significant differences between specific pairs of groups.

The results were to be interpreted in the following fashion. If a treatment group changed in a measure over time, the subsequently “improved” measure noted at the final visit would need to exceed the measure of the naïve no exposure group to represent a true difference (improvement) over expected variability across a group of “untrained” individuals.

Additionally, relationships between psychological factors (CES-D, GAD-7, Positive Affect, Negative Affect, FPQ, and Expectations) and CPM protocol were assessed using two-way analyses with Intervention (HE, LE, and NE) and Time (pre-intervention, post-intervention) as factors, controlling for age as a covariate. For variables violating normality assumptions (CES-D, GAD-7, Positive Affect, Negative Affect, and Expectations), age effects were controlled by residualizing the dependent variables (regressing out age) prior to conducting Aligned Rank Transform (ART) ANOVA. For FPQ, which met parametric assumptions, age was included directly as a covariate in a traditional ANCOVA framework, allowing for direct examination of the age covariate effect.

In our study, we included age as a covariate, given evidence that CPM efficiency declines with advancing age (53, 54). In addition, given the differences in water temperature used for men and women in the CPM we tested whether sex was associated with change in CPM by two-way ANOVA examining CPM change scores with Intervention (HE, LE) and Sex (male, female) as factors. This showed no main effect of sex [F(1,36) = 1.13, *p* = 0.295, *η*^2^ = 0.029] or Sex × Intervention interaction [F(1,36) = 1.40, *p* = 0.245, *η*^2^ = 0.036] indicating similar training effects for males and females. Similarly, no significant sex effects or interactions were observed for other QST and psychological measures; all *p* > 0.05). Therefor sex, was not included in the final models.

The adjusted significance thresholds were *p* < 0.01 for Heat Tests (5 variables: heat threshold temperature, heat threshold rating, heat tolerance temperature, heat tolerance rating, and after sensations) and *p* < 0.025 for Pressure Tests (2 variables: CPM efficiency and pressure pain threshold upper extremity).

## 4 Results

### 4.1 Participants

**Figure 4:**
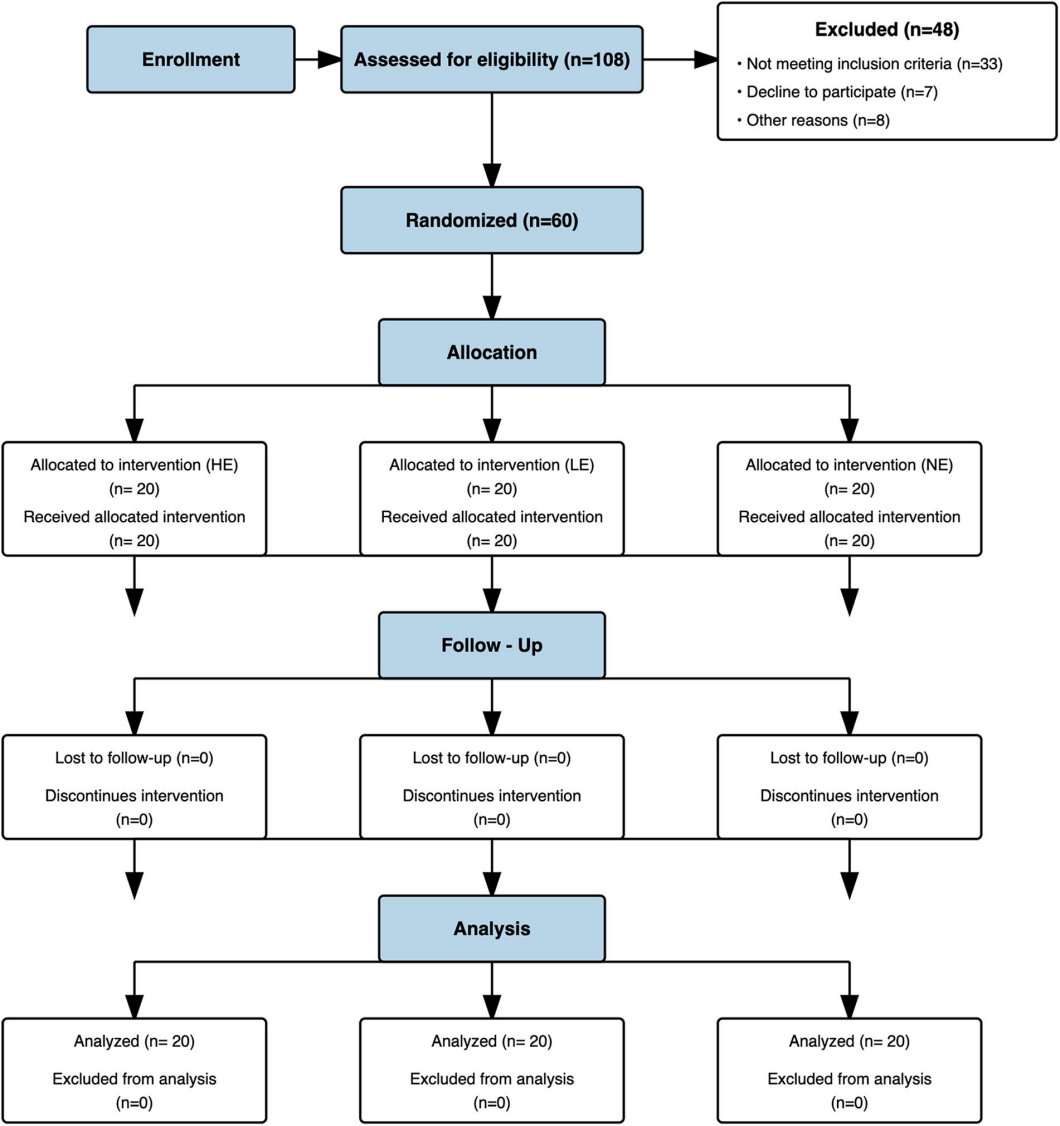
Recruitment flow chart.

Sixty pain-free individuals were enrolled into the study, with an average age of 37 ± 12.88 years (range 18 to 75 years; See Figure 4). All 60 participants confirmed pain-free status at baseline (BPI-1 = 0), meeting inclusion criteria. The participants completed a standard intake form that included gender, age, employment status, marital status, educational level, and health history including a self-report item characterizing pain tolerance relative to others as shown in Table 1.

**Table 1:** Demographic factors for the entire sample.

Participants	Mean (SD)
Participant Characteristics (*n* = 60)	
Age (years)	37.7 (12.88)
Weight (lb)	166.23 (41.50)
Height (inches)	69.32 (19.50)
Marital Status, *n* (%)	
Single	25 (41.67)
Married	31 (51.67)
Separated	1 (1.7)
Divorced	2 (3.3)
Widowed	1 (1.7)
Ethnicity, *n* (%)	
Hispanic or Latino	10 (16.67)
Not Hispanic or Latino	50 (83.33)
Race, *n* (%)	
Asian	26 (43.33)
White	21 (35)
Black or African American	9 (15)
Other Races or Multiple Races	4 (6.67)
Sex, *n* (%)	
Male	29 (48.33)
Female	31 (51.67)
Smoking Status, *n* (%)	
Non—Smoker	57 (95)
Current Smoker	3 (5)
Packs per day	0.5
Drinking Status, *n* (%)	
Non-Drinker	48 (80)
Current Drinker	12 (20)
Average drinks per week	3.3
Education Level, *n* (%)	
High School or GED	7 (11.67)
Bachelor's degree	15 (25)
Master's degree	21 (35)
Doctorate or Post Professional Degree	11 (18.33)
Associate degree	6 (10)
Employment Status, *n* (%)	
Full time	25 (41.67)
Part time	9 (10)
Student	21 (35)
Unemployed	2 (3.33)
Retired	3 (5)
Dominant Hand, *n* (%)	
Right	59 (98.33)
Pain Tolerance Characterization, *n* (%)	
Relatively the same as other people, I can tolerate moderate amounts of pain	26 (43.33)
I don't know how much pain I can tolerate	10 (16.67)
Higher than most people I know, I can tolerate a high amount of pain	21 (35)
Lower than most people I know, I can tolerate low amounts of pain, but not high	3 (5)

SD, standard deviation; n, number.

Psychological characteristics collected at the baseline of the total sample are presented in Table 2. An ANOVA was used to compare participants in the three different arms on baseline psychological characteristics. Groups did not differ on the psychological factors (*p*'s > 0.05) at baseline. All participants scored below clinical cut-offs on psychological measures. Nor did groups differ on the proportion of men and women in each group [*χ*^2^(2) = 0.87, *p* = 0.65]. No adverse events related to pain assessment or intervention were reported by any of the subjects during or after the study.

**Table 2:** Baseline psychological variables for the total sample.

Psychological Variables	M	SD
CES-D	11.61	9.70
GAD7	3.64	3.99
NA	17.94	6.60
PA	35.15	7.9
FPQ-9	22.21	6.85
Anxiety about Pain Testing	21.93	24.38
Expectations	10.41	5.58

M, mean; SD, standard deviation; CES-D, center for epidemiological studies—depression scale; GAD7, generalized anxiety disorder-7; NA, negative affect; PA, positive affect; FPQ, fear of pain q.

### 4.2 Conditioned pain modulation efficiency

**Figure 5:**
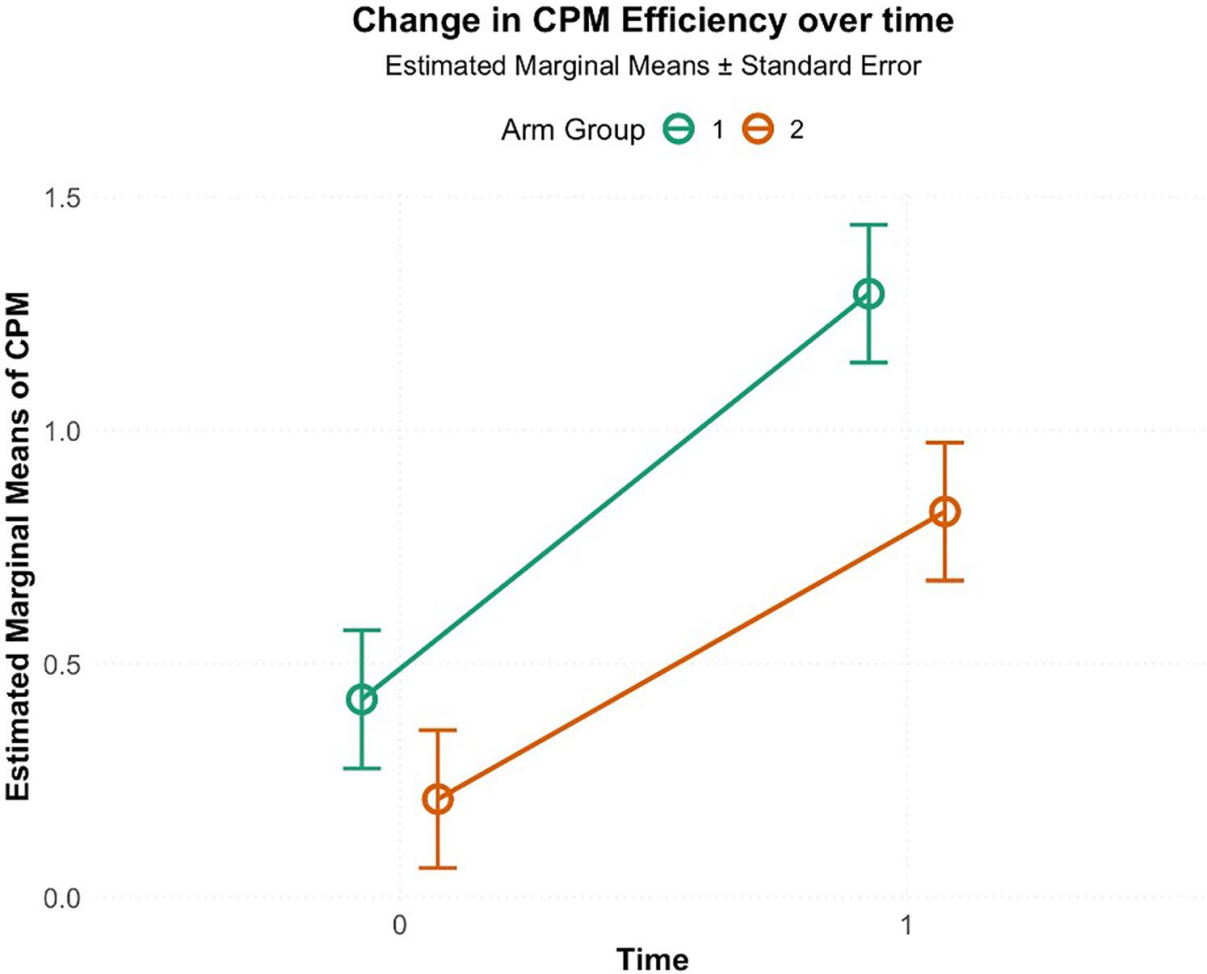
Change in CPM efficiency from pre-intervention (0) to post-intervention (1; 5th visit) in high-exposure (Arm 1) and low-exposure (Arm 2) groups. Circles indicate estimated marginal means, and error bars represent standard errors. Both groups showed increased CPM efficiency, with greater improvement in the high-exposure group.

A two-way ANOVA was conducted to examine the effect of intervention type (HE or LE) and time (pre-intervention, post-intervention) on CPM efficiency as shown in Figure 5 The interaction between arm and time was not significant, F(1, 76) = 0.73, *p* = 0.39, *η*^2^ = 0.006. However, there was a significant effect for time F(1, 76) = 25.1, *p* < 0.001, *η*^2^ = 0.23, indicting an overall improvement in CPM efficiency across the participants. There was also a significant effect for intervention, F(1, 76) = 5.31, *p* = 0.02, *η*^2^ = 0.05, indicating that participants in HE had higher average CPM efficiency (M = −0.87, SD = 0.20) compared to LE (M = −0.61, SD = 0.20).

**Figure 6:**
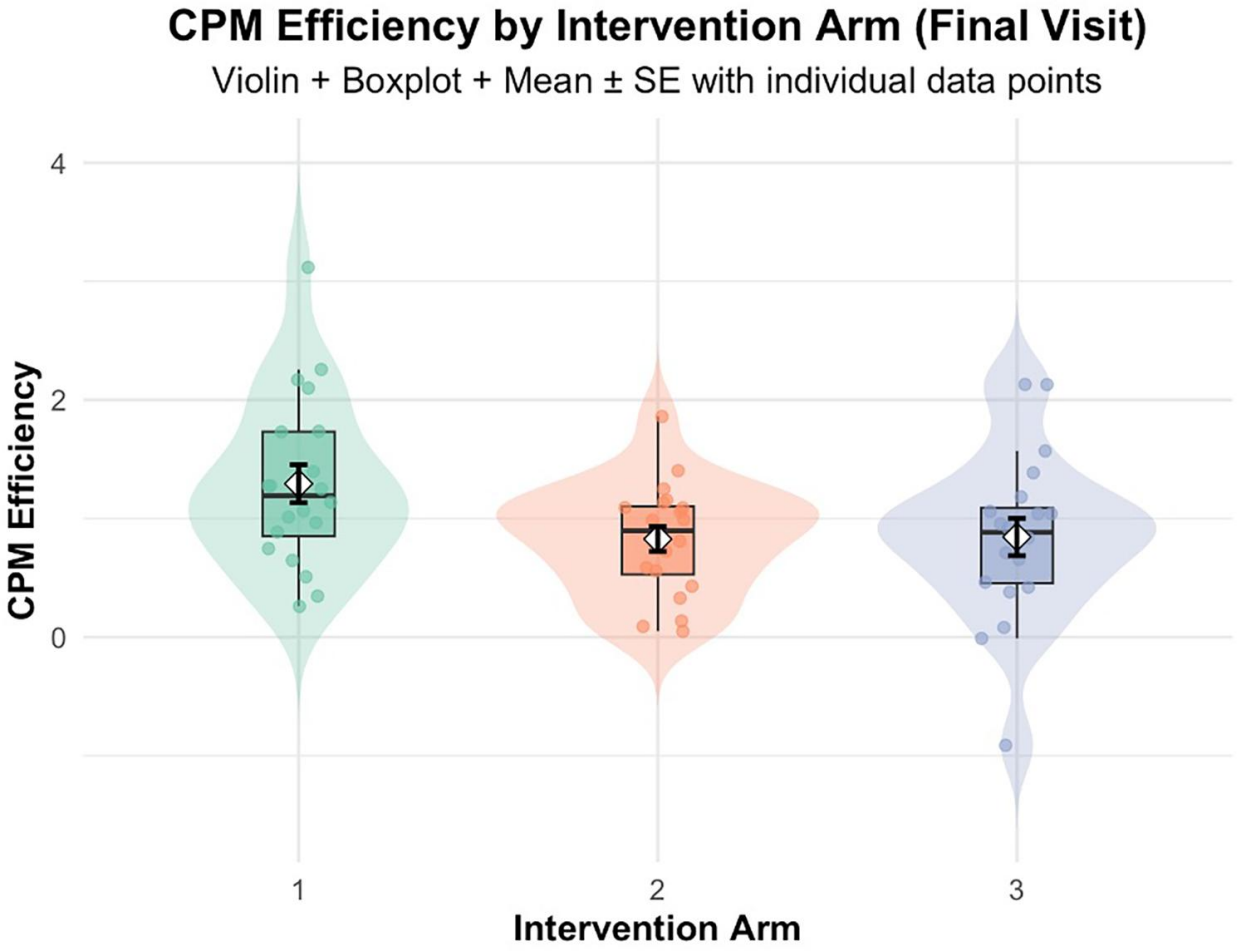
CPM efficiency at final visit by intervention arm: Arm 1 (high exposure), Arm 2 (low exposure), Arm 3 (no exposure/control). Violin plots depict data distribution; boxplots show medians and interquartile ranges; diamonds indicate means ± SE; individual data points are overlaid.

The follow up one-way ANOVA indicated an intervention (HE, LE and NE) effect on CPM efficiency [F(2, 57) = 3.42, *p* = 0.030, *η*^2^ = 0.107], indicating an overall difference in the group means. *Post-hoc* analyses showed effect sizes for the difference between HE and LE (Cohen's *d* = 0.730, *p* = 0.053), and between HE and NE (Cohen's *d* = 0.702, *p* = 0.060), and very small effect size for the difference between LE and NE (Cohen's *d* = 0.028, *p* = 0.996). Estimated marginal means showed that HE had the highest CPM efficiency (M = 1.29, SE = 0.14), followed by LE (M = 0.84, SE = 0.14) and NE (M = 0.83, SE = 0.14) as shown in Figure 6. Complete ANOVA results are provided in Supplementary Table S1.

### 4.3 Pain sensitivity (QST)

Heat threshold ratings showed a significant main effect for intervention [F(1,76) = 12.11, *p* < 0.001, *η*^2^ = 0.13] when comparing HE and LE groups. Between-group comparisons at final visit revealed significant differences for heat threshold ratings [*χ*^2^(2) = 10.615, *p* = 0.004]. All other QST measures, including heat threshold temperature, heat tolerance (temperature and rating), after sensations, and pressure pain threshold, showed no significant effects (all *p* > 0.05). Complete statistical details for all QST measures are provided in Supplementary Tables S2–S4.

To examine whether improvements in heat threshold ratings were associated with psychological factors, we conducted correlation analyses between change scores (post-intervention minus pre-intervention) for heat threshold ratings and changes in all measured psychological variables (CES-D, GAD-7, Positive Affect, Negative Affect, FPQ, and Expectations). No significant correlations were observed between changes in heat threshold ratings and any psychological factor (all *p* > 0.05), suggesting that the observed improvements in pain intensity ratings at threshold were independent of changes in psychological state.

### 4.4 Psychological factors

No significant main effects of intervention, time, or their interactions were observed for any psychological variables: GAD-7, Positive Affect, Negative Affect, CES-D, Expectations, or FPQ (all *p* > 0.05, with consistently small effect sizes *η*^2^ < 0.05). Complete results are provided in Supplementary Table S5.

## 5 Discussion

This study examined the potential for repeated activation of endogenous inhibition, using a specific CPM protocol as a “training” stimulus, to modify pain sensitivity and improve endogenous inhibition as measured by CPM. Our results were mixed in this regard.

First, these results provide preliminary support for the primary hypothesis that repeated exposure to a training stimulus based on CPM would improve CPM measured with different modalities. The two-way ANOVA demonstrated significant improvement in CPM efficiency over time in intervention groups (HE and LE; *p* < 0.001), with a significant intervention effect (*p* = 0.02) indicating HE outperformed LE. Comparisons at the final visit using data from the unexposed NE group revealed important nuances. While the one-way ANOVA indicated an overall intervention effect across all three groups [F(2,57) = 3.42, *p* = 0.030, *η*^2^ = 0.107], *post-hoc* comparisons between HE and both LE (*p* = 0.053, Cohen's *d* = 0.73) and NE (*p* = 0.060, Cohen's *d* = 0.70) approached but did not reach conventional statistical significance. These findings should be interpreted cautiously, as the marginal *p*-values may reflect substantial between-subject variability in CPM responses, which is well-documented in the literature (8, 10). The convergent evidence from significant time and intervention effects in the two-way ANOVA, combined with large effect sizes in final visit comparisons, suggests that higher-frequency CPM exposure produces meaningful enhancements in CPM efficiency than a low exposure (1 tests of CPM and one CPM intervention) and no exposure. These preliminary findings warrant replication with larger samples to definitively establish clinical significance. The absence of significant differences in CPM efficiency between LE and NE groups (Cohen's *d* = 0.028, *p* = 0.996) suggests that low exposure to activation of CPM may be insufficient to meaningfully enhance endogenous pain inhibition beyond observed levels in unexposed individuals.

The significant improvement in CPM efficiency over time that was identified is consistent with findings from various exercise studies, highlighting the complex interplay between exercise, pain perception, and endogenous pain modulation mechanisms (24, 29, 55). While our study did not involve exercise interventions, we draw a mechanistic parallel between our findings and exercise-induced adaptations based on shared neurophysiological pathways. Conditioned pain modulation (CPM) and exercise-induced hypoalgesia (EIH) share common neurophysiological mechanisms involving endocannabinoid, opioid, and serotonergic systems (28, 29). Both paradigms activate descending inhibitory pathways through nociceptive stimulation EIH through exercise-induced nociception and CPM through heterotopic noxious conditioning. Athletic populations exhibit elevated pain thresholds attributed to repetitive nociceptive exposure during training (25), suggesting that repeated activation of pain modulatory circuits, regardless of the specific stimulus type (exercise vs. CPM), may induce neuroplastic adaptations.

Exercise-induced nociception serves as a conditioning stimulus for conditioned pain modulation (CPM) pathways, exemplified by repeated maximal handgrip exercises used in CPM studies (56, 57). Mechanistically, isometric exercise activates Group III (A-delta) and Group IV (C) afferents, stimulating endogenous pain inhibitory systems (55). Cross-age studies demonstrate CPM's predictive value for EIH (24, 29, 58), with documented pressure pain rating reductions post-exercise across age groups (24). Further, decreased CPM following isometric exercise in individuals demonstrating systemic EIH supports overlapping neurophysiological mechanisms between CPM and EIH (28).

Previous work suggests that sustained physical activity induces significant modifications in baseline pain perception, with athletic populations demonstrating elevated pain thresholds and augmented pain tolerance compared to sedentary controls (25). These adaptations likely result from repeated exposure to nociceptive stimuli during intensive training. Reduced CPM responsiveness observed in athletes may reflect compensatory neuroplastic changes resulting from chronic engagement of descending pain inhibitory pathways during high-intensity training (25). Such chronic stimulation potentially induces structural and functional modifications within central pain-processing regions, notably the thalamus and periaqueductal gray matter (59). Consequently, the observed enhancement in CPM efficiency in the current investigation may be attributed to systematic activation of descending pain inhibitory pathways through repeated activation of CPM, analogous to exercise-induced adaptations in pain sensitivity. However, we acknowledge this comparison is theoretical, and the specific mechanisms underlying CPM training effects may differ from those of exercise-induced adaptations.

The other behavioral estimate of inhibition evaluated in the current study, aftersensations, did not show significant changes in intensity reported or the rate at which these sensations decayed. This is contrary to what we had hypothesized as aftersensations and the rate at which they decay are also considered to reflect endogenous inhibitory mechanisms. A limitation of the current study that may explain the lack of aftersensation changes is the inclusion of pain-free individuals who where unlikely to have the impaired inhibitory mechanisms identified in people with chronic pain conditions such as fibromyalgia (39, 60) that are associated with prolonged after sensations from QST.

In addition, the present study also evaluated the potential relationship between repeated activation of CPM and static measures of pain sensitivity, including heat threshold and tolerance. Statistical analyses demonstrated that participants in the High Exposure group exhibited significant alterations in their heat threshold ratings compared to the Low Exposure group and No Exposure groups. These findings provide further evidence for changes in pain inhibition capabilities after training. However, other pain sensitivity parameters, including heat threshold temperatures, heat tolerance ratings, and pressure pain thresholds in the upper extremity, remained statistically unchanged between the intervention groups. The selective change in heat threshold ratings, while threshold temperatures remained constant, suggests central modifications in pain signal evaluation at the threshold level. Participants detected pain at the same stimulus intensity (unchanged threshold temperatures) but rated these newly-painful threshold stimuli as less intense (improved threshold ratings). This indicates enhanced descending inhibitory modulation of pain intensity coding specifically at the pain detection threshold, rather than affecting suprathreshold pain processing or primary nociceptor sensitivity.

However, the specificity of CPM enhancement observed raises intriguing mechanistic questions. Although CPM efficiency significantly improved, this effect did not generalize to other pain measures such as heat tolerance or aftersensations, despite these being traditionally viewed as suprathreshold pain indicators. This specificity poses a fundamental inquiry: why would activation of descending inhibitory pathways via CPM selectively enhance CPM itself but not broader pain modalities?

Several explanations may account for these selective findings. First, the findings may reflect task-specific neural network adaptations within specialized pain processing circuits. Pain processing involves multiple, functionally distinct neural networks that can undergo independent plastic modifications (61–63). The repeated activation of CPM-specific circuits involving complex integration between ascending nociceptive signals, descending modulatory pathways, and cognitive-evaluative networks may have induced selective synaptic strengthening within these specialized networks (64, 65). This hypothesis suggests that different pain modulation paradigms engage distinct neural assemblies with limited cross-talk. The temporal dynamics of CPM, requiring rapid integration of spatially separated nociceptive inputs, may depend on specialized interneuronal circuits that remain functionally isolated from networks mediating heat tolerance or pressure pain processing (66, 67).

Second, procedural learning effects that extend beyond simple task familiarity. Participants may have developed complex cognitive-perceptual strategies specifically optimized for managing dual-stimulus paradigms. Our sequential CPM paradigm requires participants to encode the initial test stimulus, maintain this representation during conditioning stimulation, and then compare it to the post-conditioning test stimulus. This learning process likely involves multiple cognitive domains, including attention allocation, expectancy management, and temporal prediction (68–70). The CPM protocol requires participants to simultaneously monitor and evaluate two distinct nociceptive inputs while comparing pain intensity changes. This complex cognitive task may benefit from practice effects that enhance attentional efficiency, improve stimulus discrimination abilities, and optimize cognitive resource allocation, all without affecting fundamental nociceptive processing mechanisms (71).

Third, neuroimmune interactions in pain processing suggests another mechanistic pathway (72–74). Repeated nociceptive stimulation can induce complex changes in microglial activation states and astrocytic function depending on stimulus characteristics (75). The specific pattern of nociceptive input during CPM training may have induced beneficial neuroimmune adaptations such as enhanced anti-inflammatory cytokine production or improved glial-neuronal communication that remain localized to the neural circuits processing CPM-type stimulus interactions (76). This mechanism could explain the specificity of our findings, as different pain paradigms may engage distinct neuroimmune signaling pathways with limited functional overlap (77). However, this mechanism remains speculative in our study and warrants direct investigation using appropriate neuroimmune biomarkers in future research.

The primary objective of this investigation was to evaluate the efficacy of repeated CPM exposure on CPM efficiency enhancement, while concurrently examining the potential association between pain sensitivity and CPM protocol. While our study demonstrates that repeated CPM exposure can enhance CPM efficiency, failure to observe concurrent improvements in other pain measures suggests that our intervention may not have activated the broad-spectrum endogenous inhibitory mechanisms typically attributed to CPM. These findings have significant implications for both research methodology and clinical applications. For researchers, our results highlight the need for comprehensive assessment batteries when evaluating interventions targeting pain inhibitory mechanisms. For clinicians, these findings suggest caution in generalizing CPM-based interventions to broader pain management contexts without additional validation.

A methodological consideration of this investigation resided in the dual implementation of CPM as both an assessment instrument and therapeutic intervention, facilitating precise quantification of neurophysiological adaptations and temporal changes in CPM efficiency. However, our use of different conditioning stimuli for assessment (heat) vs. intervention (cold water) introduces variability, as CPM efficiency is known to be highly dependent on specific protocol parameters (1, 8)The recruitment of healthy, pain-free participants enabled the isolation of intervention effects on neural mechanisms without confounding pathological variables, enhancing methodological rigor and result reliability.

Successful CPM response induction aligned with *a priori* hypotheses, given the neurotypical participant cohort. The experimental paradigm employed validated cold water stimulation protocols for CPM response elicitation, as previously established by Ibancos-Losada et al. (33). The implementation of Pain 40 as a stimulus for Pressure Pain Threshold (PPT) determination was supported by prior research demonstrating its efficacy in CPM response induction (8). The selected stimulus intensity demonstrated sufficient magnitude for CPM response elicitation, while anatomical testing sites (dominant arm and contralateral foot) aligned with established research indicating minimal site-dependent variations in CPM response, excepting ipsilateral and homotopic regions (78).

Methodological limitations warrant consideration in result interpretation. The exclusive recruitment of painfree participants limits generalizability to chronic pain populations, necessitating future validation in clinical cohorts with compromised CPM efficiency. This cohort may also have different responses to people with chronic pain conditions; for example, lower aftersensations. Additionally, this study limited to a two-week protocol without extended follow-up. This prevents assessment of whether CPM efficiency enhancements persist over time or diminish after intervention cessation. Furthermore, our use of different conditioning stimuli for assessment (heat) vs. intervention (cold water) introduces variability, as CPM efficiency is known to be highly dependent on specific protocol parameters (1, 8). Though we cannot rule out potential variability in training effects based on individual stimulus parameters, this design choice was necessary to minimize practice effects between assessment and intervention protocols. Our analytical approach has a limitation was the post-test-only design for the NE control group, precluding the determination of whether observed improvements in the HE group exceeded natural history or practice effects. Although HE demonstrated superior post-intervention CPM efficiency compared to both LE and NE groups, the absence of longitudinal control data prevents definitive attribution of improvements to repeated CPM exposure vs. test-retest reliability, learning effects, or natural variation. Future studies should incorporate pre-post assessments for all groups to isolate intervention effects from time-related changes.

Future research directions should encompass investigation of additional mechanistic pathways contributing to observed outcomes, alongside modification of CPM interventions and incorporation of comprehensive physiological measures. Extended intervention periods beyond the current two-week protocol may be necessary for optimal CPM efficiency enhancement, suggesting the importance of longitudinal studies examining neuroplastic adaptation timeframes. While the current methodology utilizing healthy participants provides foundational insights, expansion to diverse pain populations is crucial for establishing clinical utility. Development of individualized CPM protocols based on pain profiles and response characteristics represents a promising therapeutic avenue, warranting further investigation.

## 6 Conclusion

In this prospective interventional investigation, we evaluated the effect of repeated activation of CPM on endogenous pain inhibitory mechanisms. We identified increases in CPM efficiency in the high exposure group that exceeded those of the low and no exposure groups. Analyses of pain sensitivity parameters (significant alterations observed in heat threshold ratings) were also identified and potentially related to the modification in endogenous modulatory capacity.

While these findings suggest promising modulatory effects on descending inhibitory pathways, the study's methodological limitation of exclusively recruiting healthy, painfree participants with presumably intact pain inhibition mechanisms necessitates cautious interpretation due to potential ceiling and floor effects. Future research directions should include chronic pain populations as well as employ advanced neuroimaging modalities (e.g., functional magnetic resonance imaging, magnetoencephalography) and comprehensive physiological measures. These additional measures to this area of study should better elucidate the underlying neural substrates and systemic adaptations associated with CPM enhancement and provide more information concerning the potential therapeutic utility and optimal implementation within existing pain management protocols.

## Data Availability

The raw data supporting the conclusions of this article will be made available by the authors, without undue reservation.
