# Supplementary material for: Impact of repeated exposure to CPM on CPM efficiency and pain sensitivity in healthy adults: a randomized controlled trial
Source: Front Pain Res (Lausanne). 2025 Nov 13;6:1677563. doi: 10.3389/fpain.2025.1677563 (PMC12657500; doi:10.3389/fpain.2025.1677563)
Supplement: Supplementary file 1 [file Table1.docx]

Supplementary Tables

Table S1. Two-way ANOVA for the relationship between CPM efficiency and CPM protocol (HE and LE)

| **Effect** | **F (df)** | **P value** | **Eta-squared** |
| --- | --- | --- | --- |
| Intervention/Arm | 5.31 (1,76) | 0.02 | 0.05 |
| Time | 25.1 (1,76) | <0.001 | 0.23 |
| Interaction (Intervention * Time) | 0.73 (1,76) | 0.39 | 0.006 |

df-Degree of Freedom

Table S2. Two-way ANOVA for the relationship between Pain Sensitivity and CPM protocol (HE and LE)

|  | **Effect** | **F (df)** | **P value** | **Eta-squared** |
| --- | --- | --- | --- | --- |
| **Two Way ART ANOVA** | | | | |
| **Heat Threshold** | Intervention/Arm | 0.30 (1,76) | 0.86 | 0.003 |
|  | Time | 0.02 (1,76) | 0.58 | 0.00026 |
|  | Interaction (Intervention * Time) | 0.24 (1,76) | 0.62 | 0.0031 |
| **Heat Threshold Rating** | Intervention/Arm | 12.11 (1,76) | <0.001 | 0.13 |
|  | Time | 1.90 (1,76) | 0.17 | 0.024 |
|  | Interaction (Intervention * Time) | 2.30 (1,76) | 0.13 | 0.029 |
| **Heat Tolerance Rating** | Intervention/Arm | 0.16 (1,76) | 0.68 | 0.002 |
|  | Time | 0.71 (1,76) | 0.40 | 0.0093 |
|  | Interaction (Intervention * Time) | 0.07 (1,76) | 0.79 | 0.0009 |
| **After Sensations** | Intervention/Arm | 0.65 (1,76) | 0.41 | 0.008 |
|  | Time | 1.10 (1,76) | 0.29 | 0.014 |
|  | Interaction (Intervention * Time) | 4.23 (1,76) | 0.04 | 0.052 |
| **Pressure Pain Threshold Upper Extremity** | Intervention/Arm | 0.30 (1,76) | 0.58 | 0.0039 |
|  | Time | 0.02 (1,76) | 0.87 | 0.0003 |
|  | Interaction (Intervention * Time) | 1.84 (1,76) | 0.17 | 0.023 |
| **Two Way ANOVA** | | | | |
| **Heat Tolerance** | Intervention/Arm | 0.06 (1,76) | 0.79 | 0.0008 |
|  | Time | 0.98 (1,76) | 0.32 | 0.01 |
|  | Interaction (Intervention * Time) | 2.27 (1,76) | 0.13 | 0.02 |

df-Degree of Freedom

Table S3. Kruskal Wallis Chi Squared test for Relationship between Final Visit QST and CPM Protocol (HE, LE and NE)

|  | **Kruskal-Wallis Chi- Squared** | **Df** | **P Value** |
| --- | --- | --- | --- |
| Arm (HE, LE and NE) | | | |
| Final Visit Heat Threshold Rating | 10.61 | 2 | 0.004 |
| Final Visit Heat Tolerance | 2.57 | 2 | 0.27 |
| Final Visit Heat Tolerance Rating | 0.62 | 2 | 0.72 |
| Final Visit After Sensations | 7.25 | 2 | 0.26 |
| Final Visit Pressure Pain Threshold Upper Extremity | 1.85 | 2 | 0.39 |

Table S4. One-Way ANOVA for the relationship between Final Visit QST and CPM Protocol (HE, LE, and NE)

|  | **Sum of Square** | **Mean Square** | **F Value** | **P Value** | **Eta-Squared** |
| --- | --- | --- | --- | --- | --- |
| Arm (HE, LE and NE) | | | | | |
| Final Visit Heat Threshold | 7.50 | 3.75 | 0.46 | 0.63 | 0.015 |

Table S5. Two-way ART ANOVA (age adjusted) and ANCOVA for the relationship between Psychological factors and CPM protocol

| **Measure** | **Effect** | **F (df)** | **P value** | **Eta-squared** |
| --- | --- | --- | --- | --- |
| **Two Way ART ANOVA (age-adjusted)** | | | | |
| **GAD-7** | Intervention/Arm | 1.12 (2,114) | 0.32 | 0.019 |
|  | Time | 0.92 (1,114) | 0.33 | 0.008 |
|  | Interaction (Intervention * Time) | 0.05 (2,114) | 0.94 | 0.001 |
| **Positive Affect** | Intervention/Arm | 0.103 (2,114) | 0.90 | 0.0018 |
|  | Time | 0.55 (1,114) | 0.45 | 0.0048 |
|  | Interaction (Intervention * Time) | 0.07 (2,114) | 0.92 | 0.0013 |
| **Negative Affect** | Intervention/Arm | 1.44 (2,114) | 0.24 | 0.0247 |
|  | Time | 0.12 (1,114) | 0.71 | 0.0011 |
|  | Interaction (Intervention * Time) | 0.05 (2,114) | 0.94 | 0.0009 |
| **CES-D** | Intervention/Arm | 1.26 (2,114) | 0.28 | 0.021 |
|  | Time | 1.59 (1,114) | 0.20 | 0.013 |
|  | Interaction (Intervention * Time) | 0.13 (1,114) | 0.87 | 0.0024 |
| **Expectations** | Intervention/Arm | 0.74 (2,114) | 0.47 | 0.013 |
|  | Time | 1.02 (1,114) | 0.31 | 0.0089 |
|  | Interaction (Intervention * Time) | 0.36 (2,114) | 0.69 | 0.0063 |
| **Two Way ANOVA** | | | | |
| **FPQ** | Intervention/Arm | 3.32 (2,114) | 0.04 | 0.05 |
|  | Time | 0.35 (1,114) | 0.55 | 0.002 |
|  | Interaction (Intervention * Time) | 0.14 (2,114) | 0.86 | 0.002 |
|  | Age (Covariate) | 0.95(1,113) | 0.33 | 0.008 |
